# Ferroptosis-Related Long Non-Coding RNA Signature Contributes to the Prediction of Prognosis Outcomes in Head and Neck Squamous Cell Carcinomas

**DOI:** 10.3389/fgene.2021.785839

**Published:** 2021-12-17

**Authors:** Wenru Jiang, Yingtao Song, Zhaowei Zhong, Jili Gao, Xiaofei Meng

**Affiliations:** ^1^ Department of Implant and Prosthodontics, The First Affiliated Hospital of Harbin Medical University, Harbin, China; ^2^ Donglai Road Stomatological Clinic, Laizhou, China

**Keywords:** head and neck squamous cell carcinoma, lncRNA, prognosis prediction, tumor immune microenvironment, immune checkpoint blockade therapy

## Abstract

**Background:** Head and neck squamous cell carcinoma (HNSCC) is a malignant tumor, which makes the prognosis prediction challenging. Ferroptosis is an iron-dependent form of non-apoptotic regulated cell death, which could affect cancer development. However, the prognostic value of ferroptosis-related long non-coding RNA (lncRNA) in HNSCC is still limited.

**Methods:** In the current study, we employed the DESeq2 method to characterize the differentially expressed ferroptosis-related genes (FEGs) between cancer and normal samples. Next, the FEG-related lncRNAs (FElncRNAs) were identified using Spearman’s correlation analysis and multiple permutation hypotheses. Subsequently, LASSO and stepwise multivariate Cox regression analyses were undertaken to recognize the prognosis-related FElncRNA signature (PFLS) and risk scores.

**Results:** Herein, we first identified 60 dysregulated FEGs and their co-expressed FElncRNAs in HNSCC. Then, we recognized a set of six FElncRNAs PFLS (*SLCO4A1-AS1*, *C1RL-AS1*, *PCED1B-AS1*, *HOXB-AS3*, *MIR9-3HG*, and *SFTA1P*) for predicting patients’ prognostic risks and survival outcomes. We also assessed the efficiency of PFLS in the test set and an external validation cohort. Further parsing of the tumor immune microenvironment showed the PFLS was closely associated with immune cell infiltration abundances. Notably, the low-risk group of the PFLS showed a higher MHC score and cytolytic activity (CYT) score than the high-risk group, implying the low-risk group may have greater tumor surveillance and killing ability. In addition, we observed that the expression levels of two immune checkpoints (ICPs), i.e., programmed cell death protein 1 (*PD-1*) and programmed cell death 1 ligand 1 (*PD-L1)*, showed significant associations with patients’ risk score, prompting the role of the PFLS in ICP blockade therapy. Finally, we also constructed a drug–PFLS network to reinforce the clinical utilities of the PFLS.

**Conclusion:** In summary, our study indicated that FElncRNAs played an important role in HNSCC survival prediction. Identification of PFLS will contribute to the development of novel anticancer therapeutic strategies.

## Introduction

Head and neck squamous cell carcinoma (HNSCC) is the sixth most common type of malignant tumor among adults with a 5-year survival rate of less than 70% (Pulte and Brenner, 2010; Johnson et al., 2020). As an epithelial tumor, HNSCC frequently appears in linings of the oral cavity, the pharynx, or the larynx (Tumino and Vicario, 2004). These tumors affect over 800,000 individuals worldwide (Johnson et al., 2020). Although novel clinical therapeutic methods have been utilized, the survival rate has not improved significantly over the recent decades (Pfister et al., 2011). Therefore, the identification of a novel signature for predicting the prognosis risk is urgently demanded.

Ferroptosis is a type of programmed cell death, which is driven by the iron-dependent peroxidation of lipids and is distinct from apoptosis, cell necrosis, and autophagy (Dixon et al., 2012; Stockwell et al., 2017; Jiang et al., 2021). Long non-coding RNAs (lncRNAs) are defined as transcripts with more than 200 nucleotides without a detectable coding potential (Wilusz et al., 2009; Ulitsky, 2016). Previous studies have demonstrated that lncRNAs showed a promising potential for indicating the tumor development risk. For example, *LINC01215* was characterized as an immune regulator and prognostic biomarker in multiple human cancers (Li et al., 2021); ferroptosis-related lncRNA *LINC00336* is highly expressed in lung cancer and acts as a competitive endogenous RNA to affect carcinogenesis (Wang et al., 2019a). However, characterization of the ferroptosis-related lncRNA signature for improving survival prediction in HNSCC is still limited.

Herein, we systemically portrayed the dysregulated FEGs between HNSCC tumors and adjacent normal tissues, including 44 up-regulated and 16 down-regulated. Next, we identified their co-expressed FElncRNAs and recognized a set of six prognosis-related FElncRNA signature (PFLS, *SLCO4A1-AS1*, *C1RL-AS1*, *PCED1B-AS1*, *HOXB-AS3*, *MIR9-3HG*, and *SFTA1P*) for predicting patients’ prognostic risks and survival outcomes (Supplementary Figure S1). Notably, the PFLS showed close associations with the tumor immune microenvironment and some immune checkpoint (ICP) expression. This highlighted the important role of PFLS in ICP blockade therapy. Finally, a drug–PFLS network was employed to identify more potential clinical utilities of PFLS.

## Materials and Methods

### Data Collection

The RNA-seq profiles and clinical information of head and neck squamous cell carcinomas (HNSCCs) were collected from The Cancer Genome Atlas (TCGA) database (https://portal.gdc.cancer.gov/). Ferroptosis-related genes (FEGs) were from the FerrDb database (http://www.zhounan.org/ferrdb/), including 111 genes that indicate the occurrence of ferroptosis, 108 genes that promote ferroptosis, and 69 genes that prevent ferroptosis (Supplementary Table S1) (Zhou and Bao, 2020). Besides, an independent HNSCC cohort GSE65858 (Wichmann et al., 2015) was obtained from Gene Expression Omnibus (GEO). The TCGA HNSCC cohort was utilized as the training cohort, while the GSE65858 dataset was employed for the external validation cohort. GSE65858 is a 270-sample HNSCC dataset yielded by the Illumina HumanHT-12 V4.0 expression beadchip (GPL10558). We employed the R package “illuminaHumanv4” to perform the probe reannotation (https://bioconductor.org/packages/release/data/annotation/html/illuminaHumanv4.db.html). For lncRNA/gene with multiple probes, the average probe intensity was used to indicate the expression level of the lncRNA/gene.

### Differential Expression Analysis of Ferroptosis-Related Genes

We identified the differentially expressed FEGs between cancer and normal samples using DESeq2 (Love et al., 2014). FEGs with adjusted *p*-values < 0.01 and $\left|\log_{2}FC\right|>1$ were considered as the differentially expressed FEGs (DE-FEGs) (Supplementary Table S2).

### Identification of Ferroptosis-Related Long Non-Coding RNAs

To identify the ferroptosis-related lncRNAs (FElncRNAs), we performed Spearman’s correlation test between the expression levels of FEGs and lncRNAs in the TCGA cohort. Spearman’s correlation *p*-values ( $P_{r}$ ) were adjusted by multiple hypotheses based on a permutation method (Yao et al., 2015). For each lncRNA, the expression value was held consistent, and in total, 10,000 random FEGs were used to perform the same Spearman’s correlation test, generating a set of 10,000 permutation *p*-values ( $P_{p}$ ). Finally, an empirical *p*-value ( $P_{e}$ ) was corrected as $P_{e}=\;\left[num\left(P_{p}\leq P_{r}\right)+1\right]/10001$ . $P_{e}$ was adjusted by the BH method ( $q_{e}$ ). LncRNAs with $q_{e}<0.001$ and an absolute value of correlation coefficient > 0.3 were identified as the FElncRNAs (Supplementary Table S3).

### Construction of the Prognosis Prediction Model

We first divided the TCGA-HNSCC dataset into three parts, with two as the training set and one as the test set. In the training, the univariate Cox regression model adjusted by gender, age, and tumor stage was constructed based on the expression levels of FElncRNAs. FElncRNAs with *p*-values < 0.05 were identified as the candidate prognosis-related FElncRNAs. Next, we employed the LASSO (least absolute shrinkage and selection operator) regression model to further screen the prognosis-related FElncRNAs and prevent the model overfitting (Supplementary Figure S2). Subsequently, we applied the bi-directional stepwise multivariate Cox regression based on the AIC (Akaike information criterion) value on the potential FElncRNAs to select the ones that minimize the AIC to attain the best model fit. A six-FElncRNA signature was identified as the prognosis-related FElncRNA signature (PFLS) which showed a significant correlation with HNSCC tumor samples’ overall survival (OS) probability. Specifically, the risk score for each patient was calculated according to the linear combination of expression values weighted by the coefficient from the multivariate Cox regression analysis:

$$Risk\;score=\sum_{k=1}^{n}coef\left(FElncRNA_{k}\right)*Expression\left(FElncRNA_{k}\right)$$

We subgrouped the samples into high-risk and low-risk groups based on the median value. of PFLS risk scores. Kaplan–Meier (KM) analysis with the log-rank test was applied to compare the survival difference between patients’ risk groups using the R package “survival”. Alternatively, the risk score was examined through the time-dependent receiver operating characteristic (ROC) curve for one-, three-, and 5-year survival, respectively. The ROC analysis was conducted using the R package “survivalROC”.

### Nomogram Analysis

We also performed the nomogram analysis to predict the one-, three-, and 5-year survival for the patients with HNSCC using the R package rms. Calibration curves were further used to assess the discrimination between actual and nomogram predicted OS probability. Besides, we adjusted other clinical features in independent prognostic analysis in order to confirm whether the PFLS was an independent indicator to predict the prognosis of patients with HNSCC.

### Tumor Immune Microenvironment Analysis

To evaluate the antitumor activity, we calculated the MHC score (Lauss et al., 2017) and cytolytic activity (CYT) score (Rooney et al., 2015). The MHC score represents the capability of antigen presentation by T cells and subsequent T cell-mediated tumor killing, which could be expressed as the average gene expression levels of the “core” MHC-I set (including genes *HLA-A*, *HLA-B*, *HLA-C*, *TAP1*, *TAP2*, *NLRC5*, *PSMB9*, *PSMB8,* and *B2M*) (Lauss et al., 2017). The CYT score indicates the cytolytic activity used by immune cells to kill tumor cells, computed as the geometric mean of the genes *GZMA* and *PRF1* (Rooney et al., 2015). We estimated the immune cell landscape using the ESTIMATE (Yoshihara et al., 2013), TIMER (Li et al., 2017), CIBERSORT (Newman et al., 2015), EPIC (Racle et al., 2017), FARDEEP (Hao et al., 2019), and MuSiC (Wang et al., 2019b; Avila Cobos et al., 2020), respectively. Next, we calculated Spearman’s correlation between the immune cell abundance and the PFLS risk score. The *p*-value was adjusted by the BH method. We also compared the immune cell abundance between high- and low-risk groups using the Mann–Whitney U test. The *p*-value was adjusted by the BH method. In addition, we performed the enrichment analysis between the risk groups and four pre-defined HNSCC microenvironment subtypes (immune-enriched, fibrotic [IE/F]; immune-enriched, non-fibrotic [IE]; fibrotic [F]; and immune-depleted [D]) by Bagaev et al*.* using accumulative hypergeometric distribution (Bagaev et al., 2021). The *p*-value was adjusted by the BH method.

### Immune Checkpoints Analysis

We manually curated 150 potential immune checkpoints (ICPs) from previous studies. And, we performed Spearman’s correlation analysis between the expression levels of these ICPs and the PFLS risk score. The *p*-value was adjusted by the BH method. Additionally, we also compared the expression levels of common immune checkpoint genes, including CD274 and PDCD1 (Waldman et al., 2020) between the high- and low-risk groups using the Mann–Whitney U test (Supplementary Table S4).

### Functional Enrichment Analysis of the Prognosis-related FElncRNA Signature

We first identified the differentially expressed genes (DEGs) between cancer and normal samples using the Mann–Whitney U test (*q*-value < 0.01, $\left|\log_{2}FC\right|>1$ ). Next, the corresponding co-expressed genes of each FElncRNA (FELGs) in the PFLS were recognized from DEGs using the permutation method mentioned above ( $q_{e}$ < 0.01, |Rho|>0.3 ). For each given FELG list, the pathway and process enrichment analysis have been carried out with the following ontology sources: KEGG Pathway, GO Biological Processes, Reactome Gene Sets, Canonical Pathways, and WikiPathways. All genes in the genome have been used as the enrichment background. Terms with adjusted *p*-values < 0.01, a minimum count of 3, and an enrichment factor > 1.5 are collected and grouped into clusters based on their membership similarities. More specifically, *p*-values are calculated based on the accumulative hypergeometric distribution, and *q*-values are calculated using the BH method to account for multiple testing. The functional enrichment analysis was conducted by Metascape (Zhou et al., 2019).

### Construction of the Drug Sensitivity Network

We obtained the FElncRNA-related drugs from the D-lnc database (Jiang et al., 2019). D-lnc included the experimentally validated and the computationally predicted modification of drugs on the lncRNA expression. And, the drug sensitivity network was constructed by Cytoscape (Shannon et al., 2003).

## Results

### Dysregulated Ferroptosis-Related Genes Were Associated With Metabolic Processes and Cancer-Related Pathways

**FIGURE 1:**
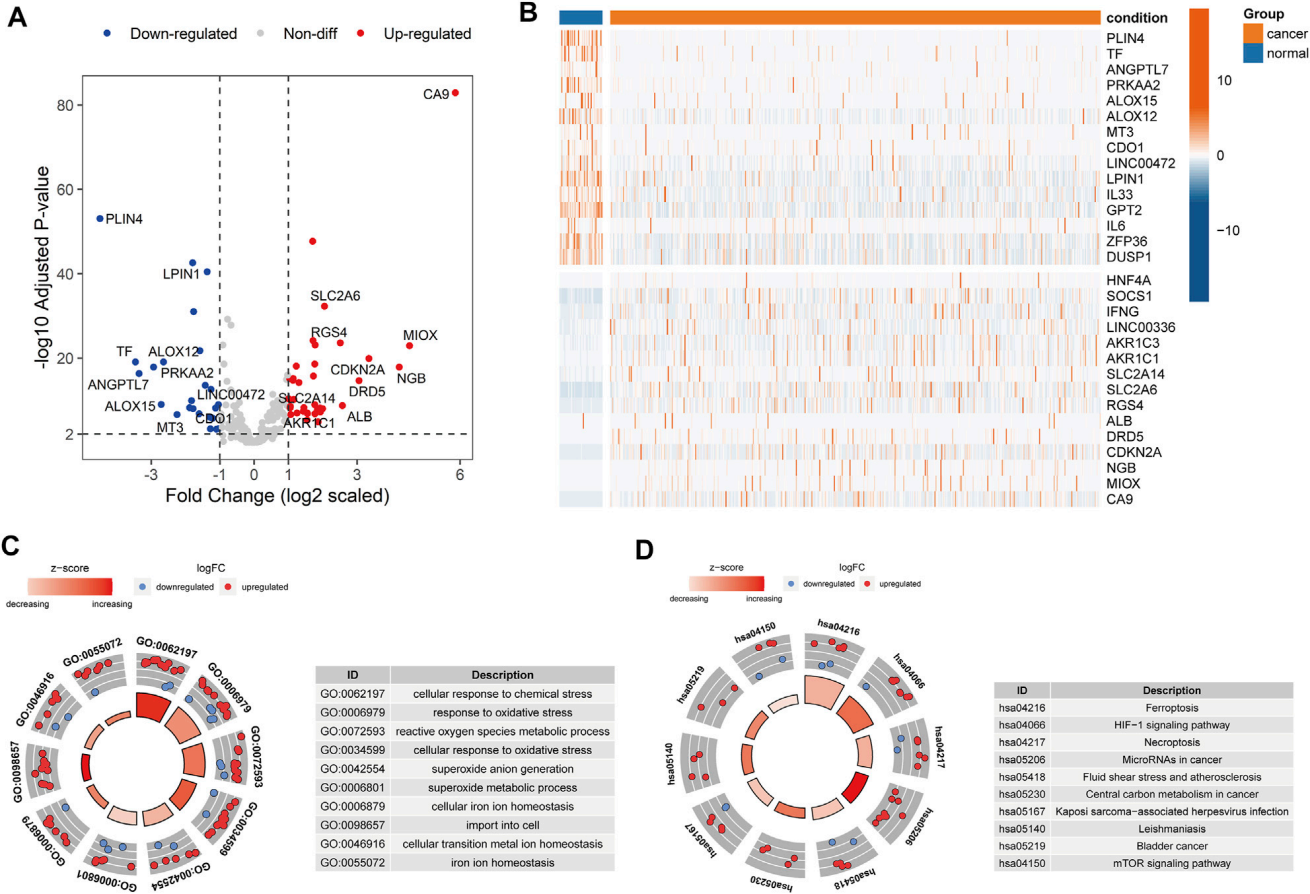
Dysregulated ferroptosis-related genes in HNSCC. **(A)** Volcano plot showing the differentially expressed FEGs between cancer and normal samples. Red and blue dots represent the up and downregulated FEGs, respectively. **(B)** Heatmap showing the top 15 up and downregulated FEGs between cancer and normal samples. C-D. Circle plot showing the z-score of significantly enriched **(C)** GO terms and **(D)** KEGG pathways. Red dots indicated upregulation, while the blue indicated downregulation. The Z-score was calculated by the R package “GOplot”. The significantly enriched GO terms and KEGG pathways are listed on the left.

To explore the expression levels of 259 ferroptosis-related genes (FEGs), we performed the differential expression analysis between cancer and normal samples in HNSCC (*Materials and Methods*). A total of 36 upregulated and 24 downregulated FEGs were identified (Figures 1A,B, Supplementary Table S2). Furthermore, we annotated their biological functions and observed that these FEGs mainly participated in multiple metabolic processes and cancer-related pathways (Figures 1C,D), such as cellular response to chemical stress (GO:0062197), reactive oxygen species metabolic process (GO:0072593), cellular response to oxidative stress (GO:0034599), microRNAs in cancer (hsa05206), and central carbon metabolism in cancer (hsa05230), etc. These results indicated that dysregulated FERGs were associated with metabolic processes and might play an important role in HNSCC.

### A Six-Ferroptosis-Related Long Non-Coding RNA Signature Contributes to the Survival Outcome Prediction

**FIGURE 2:**
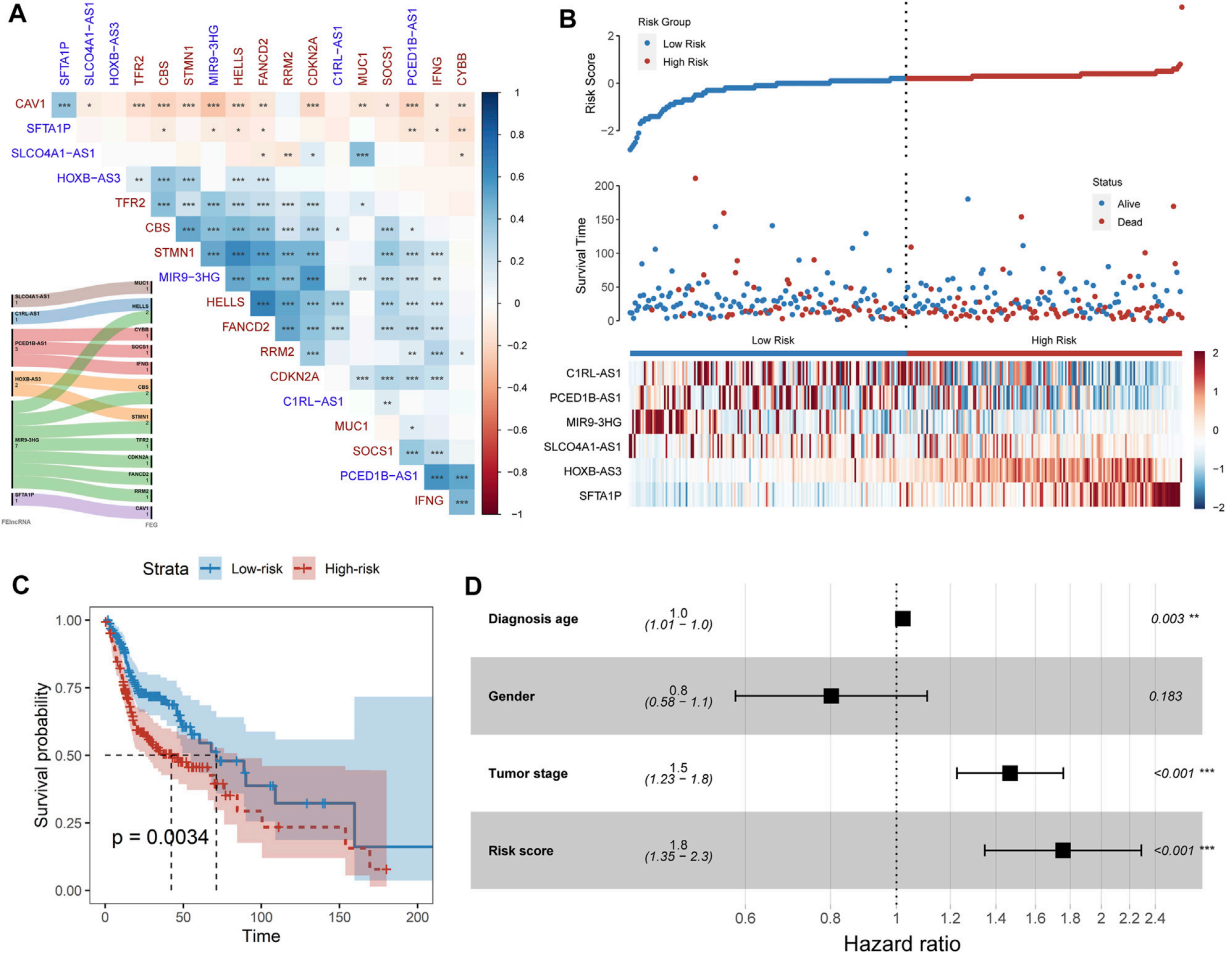
FElncRNAs indicated the prognosis values in HNSCC. **(A)** Heatmap showing the Spearman’s correlation between FElncRNAs and FEGs. **(B)** Risk score distribution, survival status, and lncRNA expression patterns for patients in high- and low-risk groups by the PFLS. **(C)** Kaplan–Meier curve of HNSCC cancer samples stratified by the risk group with the log-rank test *p*-value provided. **(D)** Forest plot showing the result of multivariate Cox regression analysis for correlation between the PFLS and clinical features and the overall survival in the TCGA cohort.

Long non-coding RNA (lncRNA) is emerging as a promising biomarker and shows prognostic values in multiple cancer types (Bolha et al., 2017; Xu et al., 2019; Yang et al., 2017). Hence, we systemically identified the ferroptosis-related lncRNAs (FElncRNAs) in HNSCC (Materials and methods). A total of 926 lncRNAs were recognized as FElncRNAs. To explore the prognosis values of these FElncRNAs, we constructed the prognostic risk model (*Materials and Methods*) and recognized a six-FElncRNA prognosis signature (PFLS). The PFLS consisted of six FElncRNAs, including two risk factors: *HOXB-AS3* and *SFTA1P*, and four protective factors: *SLCO4A1-AS1*, *C1RL-AS1*, *PCED1B-AS1*, and *MIR9-3HG*, which showed correlations with the HNSCC tumor samples’ overall survival (OS) probability (Supplementary Figure S3). We further explored the associations between the PFLS, FElncRNAs, and FEGs (Figure 2A). *MIR9-3HG* showed a broad correlation with multiple FEGs, such as *STMN1*, *CBS*, and *HELLS*. Previous studies also further supported that *MIR9-3HG* was a prognosis-related biomarker in HNSCC (Hu et al., 2020; Guo et al., 2021).

### Prognosis-Related FElncRNA Signature Reveals the Potential Prognostic Risk in Head and Neck Squamous Cell Carcinomas

We next calculated the risk score of the PFLS to characterize the prognosis risk of TCGA HNSCC. The PFLS risk score could be expressed as: (−0.423*SLCO4A1-AS1) + (−0.393*C1RL-AS1) + (−0.207*PCED1B-AS1) + (0.159*HOXB-AS3) + (−0.231*MIR9-3HG) + (0.036*SFTA1P) (Figure 2B). We then subgrouped the patients into high- and low-risk groups based on the median values of the risk score. The PFLS risk group was able to indicate patients’ survival outcomes (Figure 2C). Notably, the multivariate Cox regression model adjusted by age, gender, and tumor stage also suggested that the PFLS risk score was an independent risk factor for predicting the overall survival outcomes of HNSCC patients (HR = 1.8, *p* < 0.001) (Figure 2D). These results suggested that the PFLS was a promising biomarker for indicating the prognosis risk of HNSCC.

### Prognosis-Related FElncRNA Signature as an Independent Factor Prompts Prognostic Risk

**FIGURE 3:**
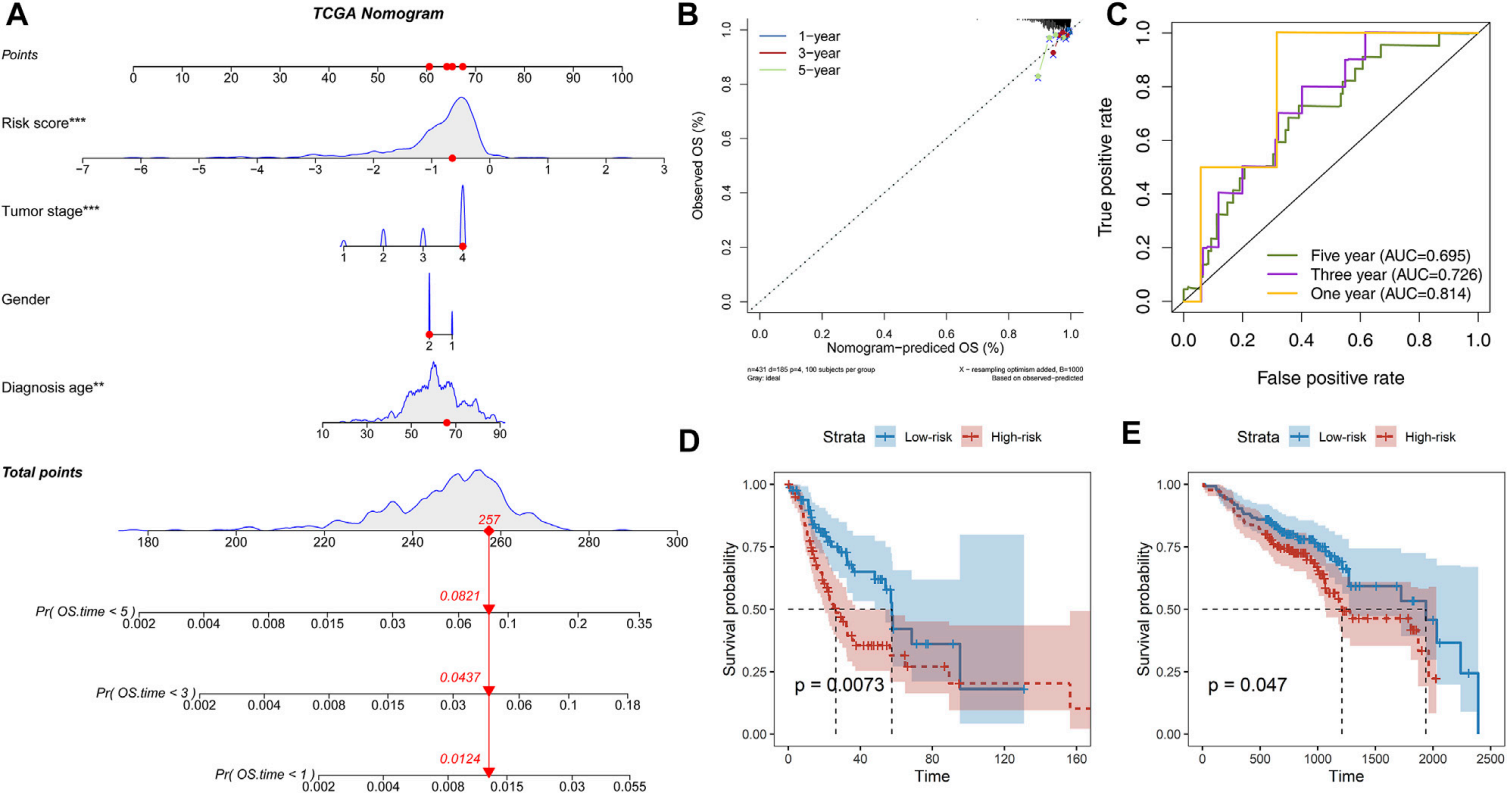
PFLS was an independent prognosis factor in HNSCC. **(A)** Nomogram composed of age, gender, stage, and risk score for the prediction of 1-, 3-, and 5-years OS probability. **(B)** Calibration for assessing the consistency between the predicted and the actual OS at 1, 3, and 5 years. **(C)** ROC curves at one, three, and five years were applied to verify the prognostic performance of the PFLS in the TCGA training set. D-E. The Kaplan–Meier curve of HNSCC cancer samples stratified by the risk group with the log-rank test *p*-value provided in **(D)** TCGA test set and **(E)** GSE65858.

Further parsing of the nomogram, PFLS was the most significant contribution to OS of one-, three-, and 5-year of HNSCC (Figure 3A). The calibration also supported that the PFLS was a promising prognostic biomarker with high accuracy (Figure 3B). In addition, the time-dependent ROC curve was constructed for one-, three-, and 5-year, and the area under the curve (AUC) was calculated to estimate the prognostic competence of the PFLS risk score (Figure 3C). The AUC of the risk score was >0.695, indicating the PFLS has powerful predictive prognostic capacity. To further confirm the PFLS is a robust biomarker in HNSCC, we employed the risk scoring model in the TCGA test set an independent HNSCC dataset GSE65858 (Wichmann et al., 2015) (*n* = 270). The PFLS risk score was identified as a poor prognosis marker (Figures 3D,E). These demonstrated the potential for PFLS risk scores to improve the prognosis of HNSCC as a complement to epidemiological features.

### Prognosis-Related FElncRNA Signature Suggests the Risk of Tumor Progression and Radiotherapy Benefits

**FIGURE 4:**
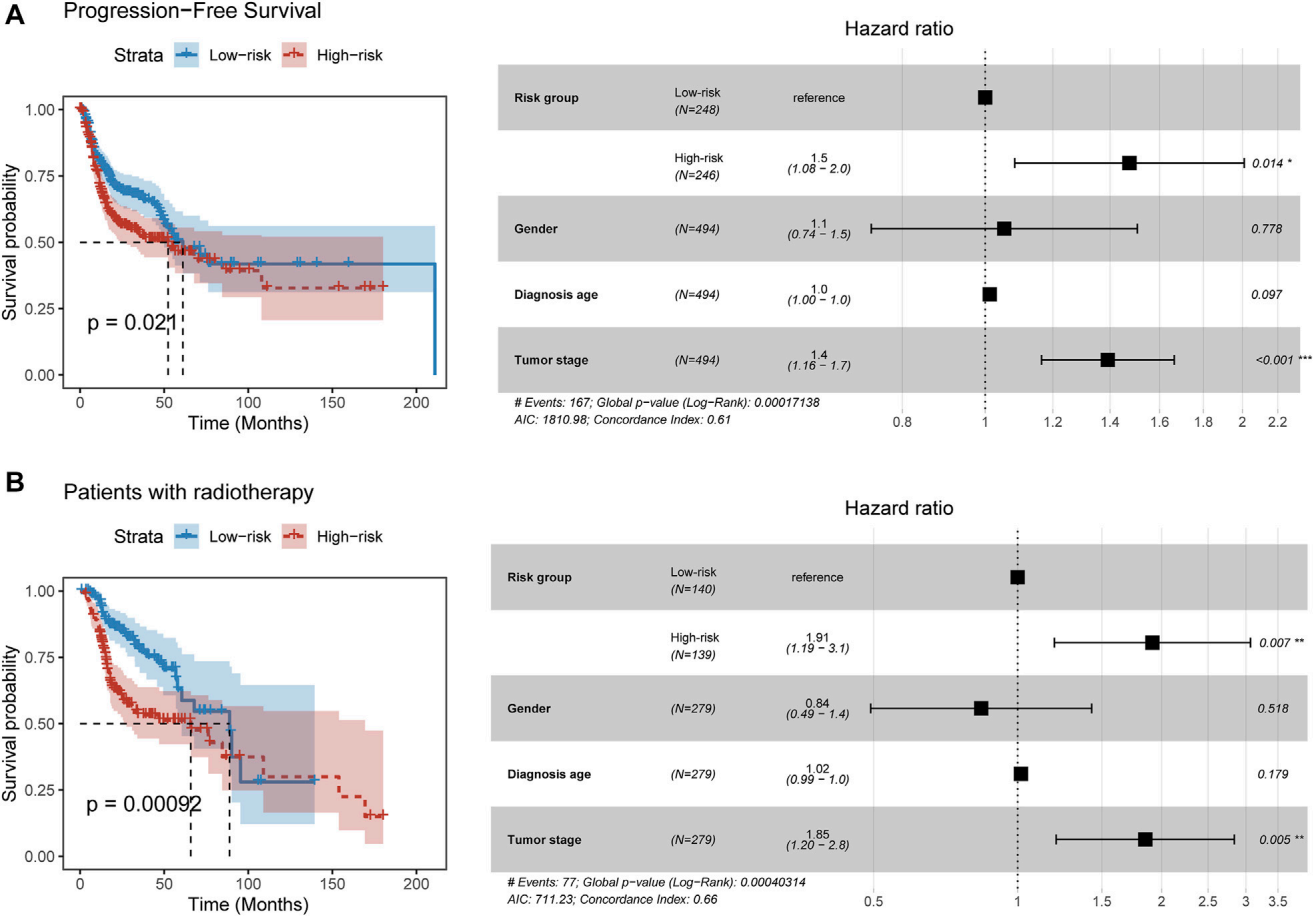
PFLS contributes to tumor progression and radiotherapy benefits. **(A,B)** On the left of each panel, the Kaplan–Meier curve of HNSCC cancer samples was stratified by the risk group with the log-rank test *p*-value provided. Forest plot showing the result of multivariate Cox regression analysis for correlation between the PFLS and clinical features and the **(A)** progression-free or **(B)** overall survival was plotted on the right.

We next explored the relationship between the PFLS and progression-free survival. By constructing the Cox proportional hazards model, we found patients in the high-risk group were more likely to experience the tumor progression than those in the low-risk group (HR = 1.5, *p* = 0.014) (Figure 4A). Furthermore, we investigated the clinical implication of the PFLS. In the patients who received radiation therapy, we found that the high-risk group of PFLS showed the association with poor OS probability (HR = 1.91, *p* = 0.007) (Figure 4B). These results also highlighted the potential clinical implications of the PFLS for predicting tumor progression and radiotherapy benefits.

### Prognosis-Related FElncRNA Signature-Risk Group Shows Association With the Tumor Immune Microenvironment and Prompts Potential Immunotherapy Values

**FIGURE 5:**
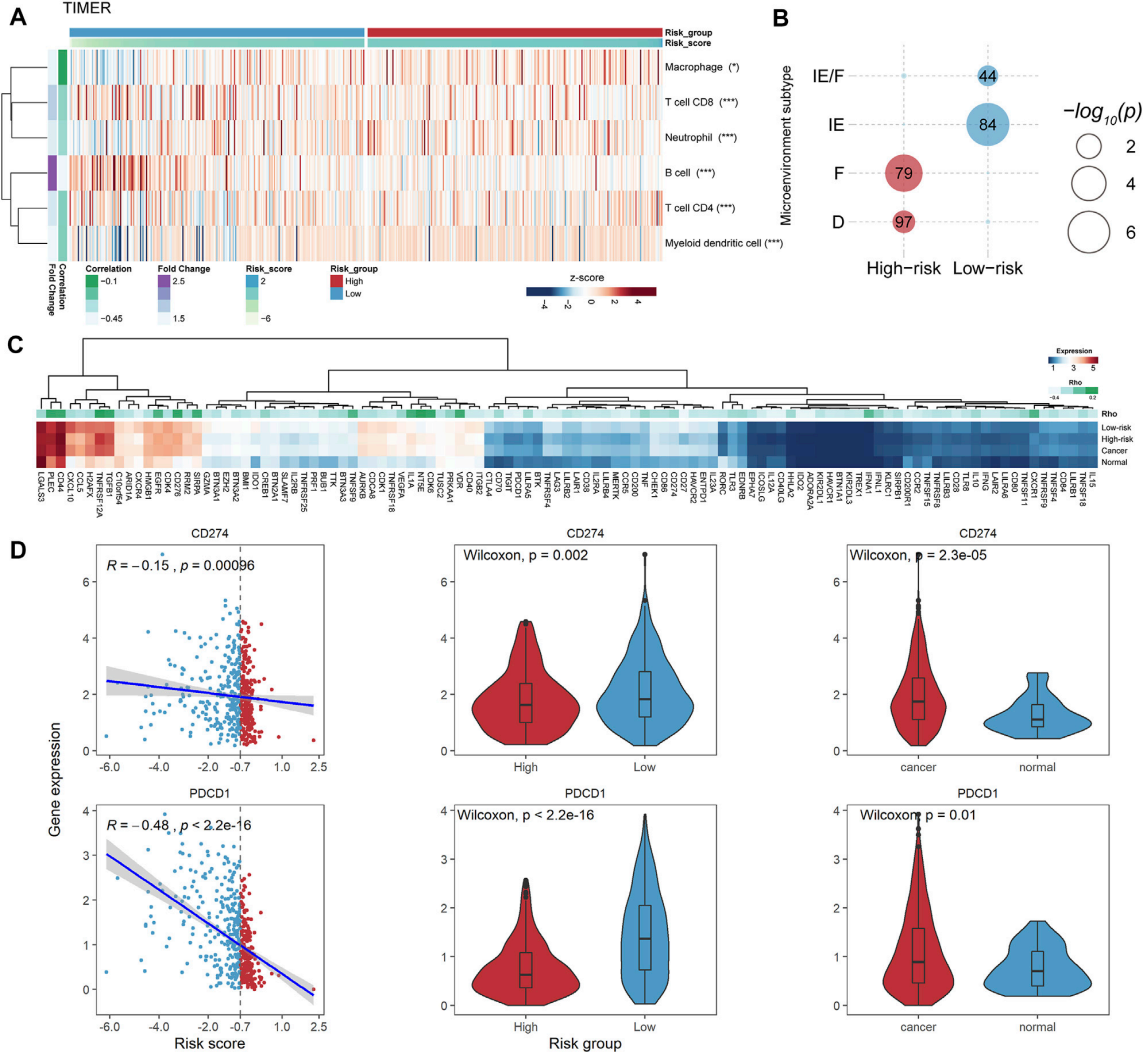
PFLS-risk groups were associated with the tumor immune microenvironment. **(A)** Heatmap showing the tumor immune cell infiltration levels assessed by TIMER. The Spearman’s correlation significance between cell abundance and the risk score was also computed for each cell shown on the right (*p < 0.05, **p < 0.001, and ***p < 0.0001). **(B)** Bubble plot showing the significant enrichment between HNSCC microenvironment subtypes and risk groups. **(C)** Heatmap showing the expression levels of PFLS-related ICPs. D. CD274 and PDCD1 were closely associated with the PFLS risk score. *p < 0.05, **p < 0.001, and ***p < 0.0001 as calculated using the **(B)** accumulative hypergeometric distribution, **(C)** Spearman’s correlation test.

To explore the potential mechanism of the PFLS in patients’ survival outcomes, we performed the gene set function enrichment based on the ferroptosis pathway (WP4313) between high- and low-risk groups. We observed the ferroptosis pathway significantly enriched in the low-risk group (Supplementary Figure S4A). Additionally, we also found that the tumor purity of the low-risk group is significantly lower than that of the high-risk group, in which the tumor immune score showed a significant difference between the two risk groups (Supplementary Figure S4B). Besides, we characterized the antitumor activity by calculating the MHC score and CYT score (*Materials and Methods*). We found that patients in the low-risk group showed higher MHC and CYT scores than those in the high-risk group (Supplementary Figures S4C,D), implying the low-risk group typically exhibited stronger tumor monitor and killing ability. Hence, we characterized the tumor immune microenvironment between the high- and low-risk groups based on multiple cell-type deconvolution algorithms (Avila Cobos et al., 2020). There were generally higher immune cell infiltration levels in the low-risk group than in the high-risk group (Figure 5A and Supplementary Figure S5), further highlighting the antitumor activity of the low-risk group.

Besides, we enriched our risk groups to four pre-defined HNSCC microenvironment subtypes (immune-enriched, fibrotic [IE/F]; immune-enriched, non-fibrotic [IE]; fibrotic [F]; and immune-depleted [D]) using accumulative hypergeometric distribution (Bagaev et al., 2021). The result showed that there were significant enrichments between the low-risk group and two immune-enriched subtypes (IE/F and IE) (Figure 5B), which could be a reasonable explanation for the better survival outcome in the low-risk group. Notably, the IE/F and IE subtypes were also characterized as the responsive factors for immune checkpoint (ICP) blockade therapy (Bagaev et al., 2021). Hence, we manually curated 150 potential ICPs from previous studies and performed Spearman’s correlation analysis between the expression levels of these ICPs and the PFLS risk score (Materials and methods). There were 109 ICPs that showed significant correlations with the PFLS risk score (Supplementary Table S4). Interestingly, we found two well-known ICPs, i.e., programmed cell death protein 1 (PD-1, *PDCD1*) and programmed cell death 1 ligand 1 (PD-L1, *CD274*), that showed obvious correlations with the PFLS risk score, which also implied the PFLS might play an important role in anti-PD-1/PD-L1 immunotherapy (Figure 5D). These results indicated that the PFLS also showed the immunological correlation and prompts the potential immunotherapy benefits.

### Biological Function Annotations for the Prognosis-Related FElncRNA Signature With Identification of Potential Clinical Implications

**FIGURE 6:**
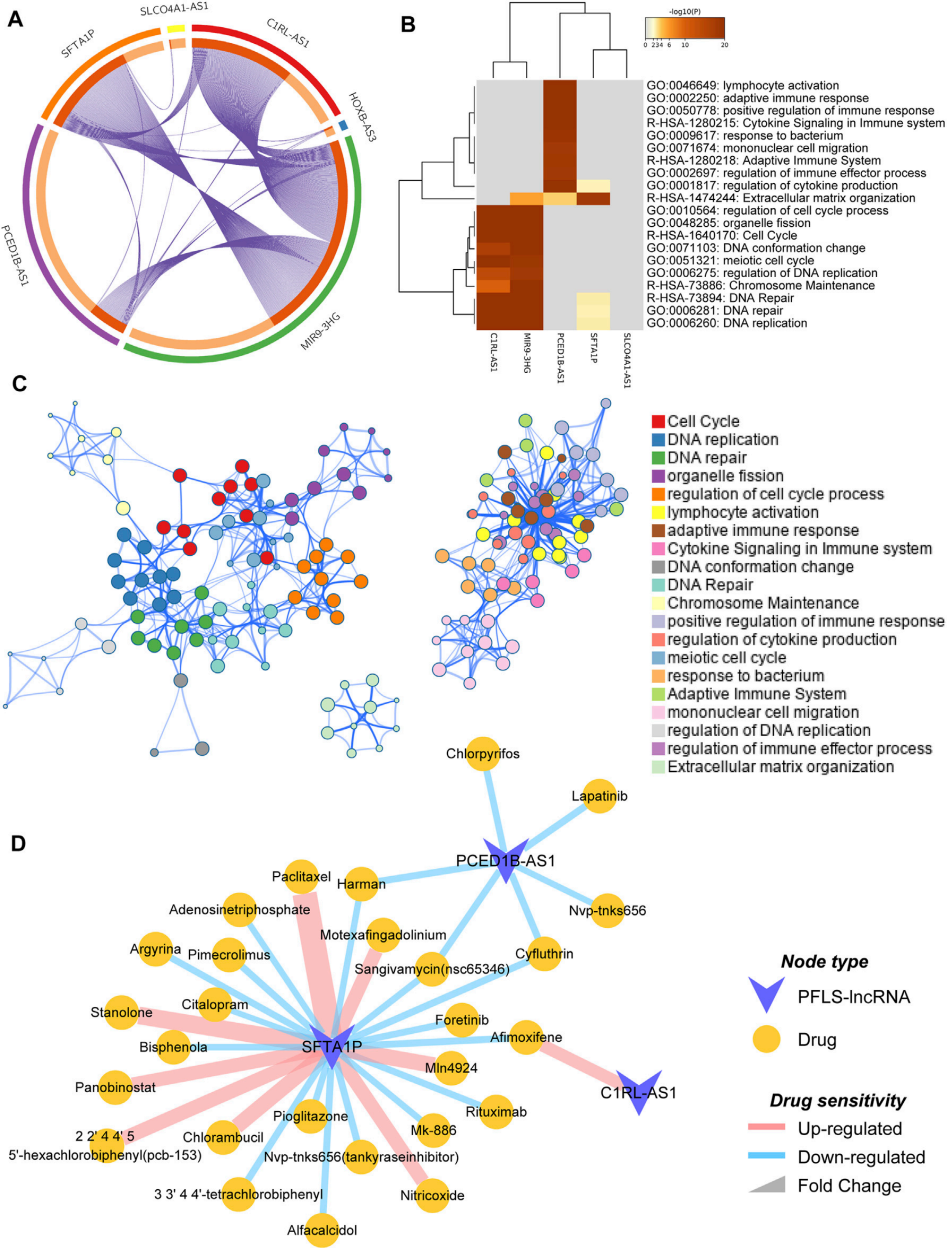
Functional enrichment analysis and potential clinical utilities of PFLS. **(A)** Circosplot showing the overlap among PFLS co-expressed genes. Purple curves link identical genes. The inner circle represents gene lists, where hits are arranged along the arc. Genes that hit multiple lists are colored in dark orange, and genes unique to a list are shown in light orange. **(B)** Network of enriched terms colored by functional clusters, where nodes that share the same functional cluster are typically close to each other. **(C)** Heatmap of enriched terms across PFLS co-expressed genes, colored by *p*-values. **(D)** PFLS-related drug sensitivity network.

To explore the potential biological functions of the PFLS, we first identified the corresponding co-expressed genes of each FElncRNA (FELGs) in the PFLS (Materials and Methods). There was obvious co-expression crosstalk among *SFTA1P*, *C1RL-AS1*, and *MIR9-3HG* (Figure 6A), implying they could share similar biological functions in HNSCC. Furthermore, based on the functional enrichment analysis, we found that PFLS could be engaged in the cell cycle , DNA replication , and immune-related biological processes (Figures 6B,C). Finally, we also constructed a PFLS drug sensitivity network to identify the potential clinical uses of the PFLS based on the drug–gene interactome data from the D-lnc database (Jiang et al., 2019). Some known anticancer drugs, such as foretinib and paclitaxel, were also contained in the network (Figure 6D and Supplementary Table S5).

## Discussion

Growing evidence has indicated that ferroptosis-related lncRNAs played an important role in tumor prognostic prediction (Lu et al., 2021; Zhu et al., 2021). However, the prognostic value of ferroptosis-related lncRNAs in HNSCC remains unknown. Hence, in this study, we explored the potential prognostic values of FElncRNAs. We first characterized the differentially expressed FEGs, which showed the biological functions manifested in multiple metabolic processes and cancer-related pathways. The result also demonstrated that ferroptosis might play an important role in HNSCC cancer development.

Next, to investigate the role of FElncRNAs in patients’ survival, we employed the stepwise multivariate Cox regression model to identify a six-FElncRNA signature named the PFLS. The PFLS showed a good performance for predicting the prognostic risk and survival outcomes in both the TCGA training set, testing, and external validation sets. Additionally, by calculating the PFLS risk score for each cancer patient, we observed that the high-PFLS risk score was a poor prognosis biomarker for the overall survival probability. These results highlighted that the PFLS was a promising biomarker with a potential prognostic value. However, compared to the wide range of HNSCC patients, our study has only covered a small proportion. Therefore, as sequencing technology becomes more widely available, we will also continue to deepen our examination of the predictive power of the PFLS for survival.

Alternatively, the PFLS also showed immunological correlations. By linking the microenvironment subtypes identified by Bagaev et al*.*, we found that the patients in the low-risk group were significantly enriched in the two immunotherapy responsive subtypes (IE/F and IE). And, we also observed that the programmed cell death protein 1 (PD-1, *PDCD1*) and programmed cell death 1 ligand 1 (PD-L1, *CD274*) showed the significant correlation with the PFLS risk score. Anti-PD-1/PD-L1 immunotherapy has emerged as an effective weapon for fighting against multiple cancer types (Waldman et al., 2020). Therefore, the correlations between PD-1/PD-L1 and PFLS risk scores could also imply the clinical immunotherapy benefits. Exploring the role of ferroptosis-related lncRNAs in the immunotherapy response will also provide new insights into the development of novel antitumor treatment strategies. In addition, by identifying the co-expressed genes and subsequent functional enrichment analysis, we observed that the PFLS mainly participated in the cell cycle, DNA repair, and immune-related biological processes. Notably, a recent study by Lin et al*.* showed that dihydroartemisinin (DHA) could induce ferroptosis and cause cell cycle arrest in head and neck carcinoma cells (Lin et al., 2016), which also highlighted the role of ferroptosis in HNSCC. Finally, we also constructed a drug–PFLS network to reinforce the clinical utilities of the PFLS. Some known and potential anticancer drugs were also included. Although these results are currently limited to the computational level, they provide a guide for our subsequent research.

In summary, our study provided a novel insight on understanding the ferroptosis-related lncRNAs in HNSCC. These FElncRNAs also showed associations with prognostic prediction, immunological associations, and potential clinical utilities.

## Data Availability

Publicly available datasets were analyzed in this study. The source code and datasets could be accessed at https://github.com/HNSCC/PFLS.

## Abbreviations

AUC, area under the ROC curve; DEG, differentially expressed gene; FEG, ferroptosis-related gene; FElncRNA, FEG-related lncRNA; HNSCC, head and neck squamous cell carcinoma; ICP, immune checkpoint; LncRNA, long non-coding RNA; OS, overall survival; PFLS, prognosis-related FElncRNA signature; ROC, receiver operating characteristic curve; TCGA, the cancer genome atlas
