# Supplementary material for: Ferroptosis-Related Long Non-Coding RNA Signature Contributes to the Prediction of Prognosis Outcomes in Head and Neck Squamous Cell Carcinomas
Source: Front Genet. 2021 Dec 17;12:785839. doi: 10.3389/fgene.2021.785839 (PMC8718757; doi:10.3389/fgene.2021.785839)
Supplement: Supplementary file 1 [file DataSheet1.PDF]

**Ferroptosis-Related Long Non-Coding RNA Signature Contributes to the Prediction of Prognosis Outcomes in Head and Neck Squamous Cell Carcinomas**

Jiang *et al*.

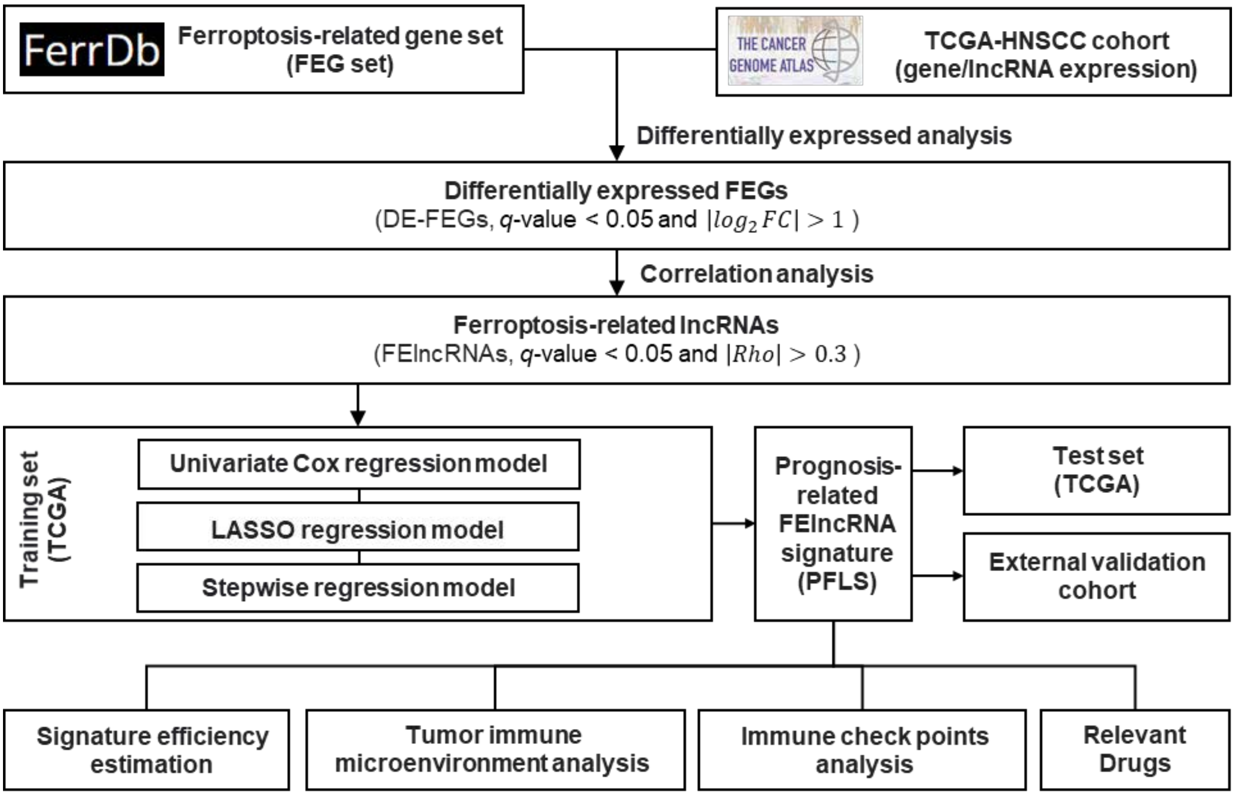


**Figure S1. Characterization of Prognosis-related FElncRNAs in HNSCC.**

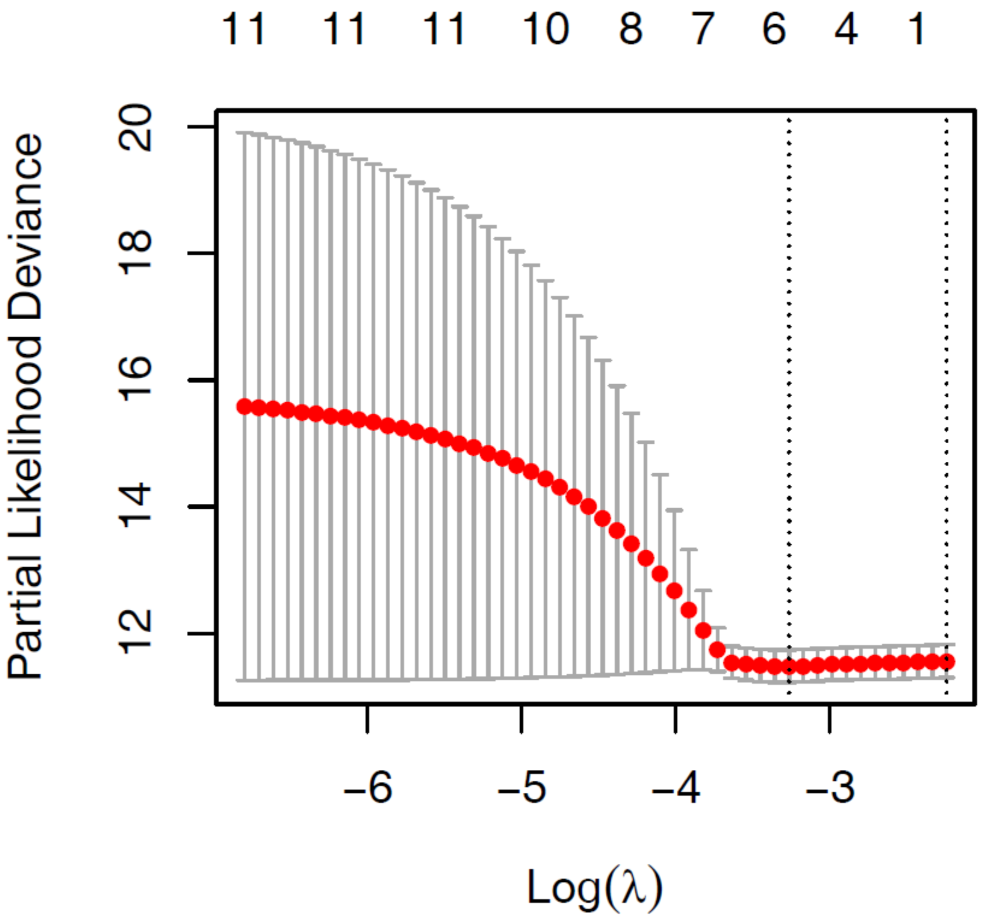


**Figure S2. The LASSO coefficients profiles of candidate prognostic FElncRNAs.**

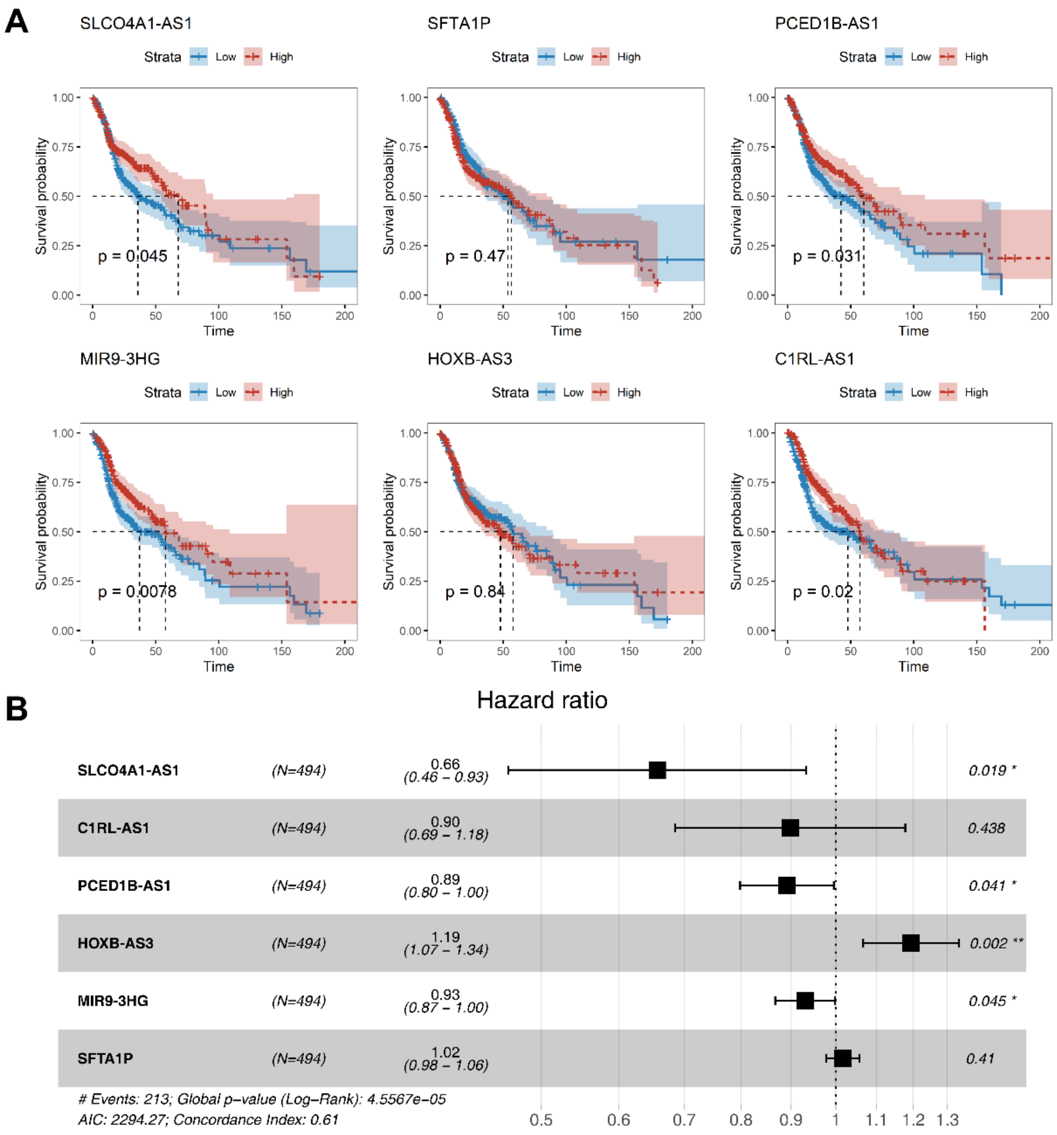


**Figure S3. Survival analysis of FElncRNAs of PFLS.** A. Kaplan-Meier curve of HNSCC cancer samples stratified by the expression of PFLS FElncRNAs with log-rank test *P*-value provided in TCGA-HNSCC cohort. B. Forest plot showing the result of multivariate Cox-regression analysis for correlation between the PFLS and the overall survival in TCGA cohort.

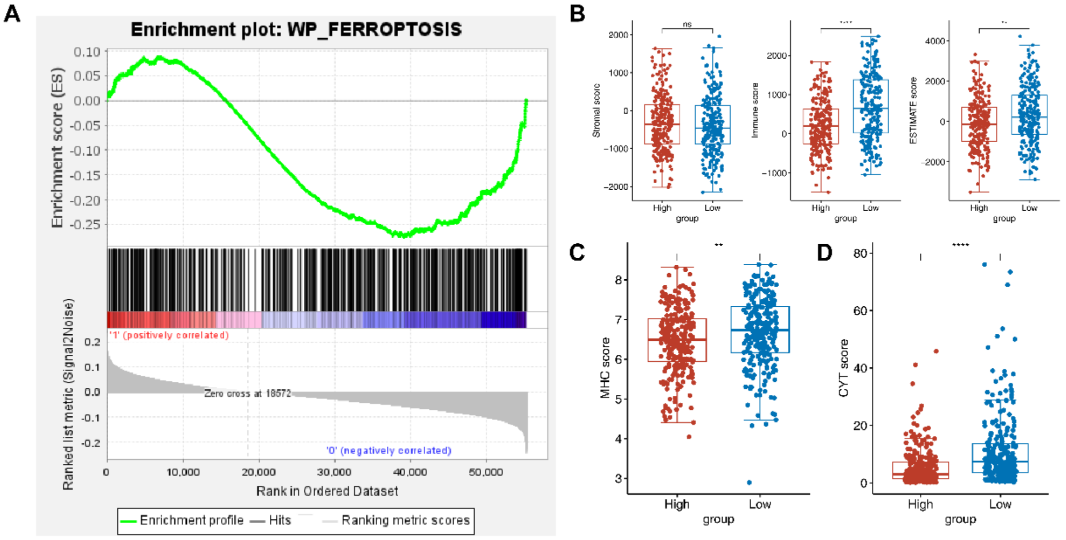


**Figure S4. Ferroptosis and immune activity in HNSCC risk group.** A. Enrichment score of ferroptosis pathway in HNSCC risk group. B. Boxplot showing the stroma score, immune score, and ESTIMATE score between low- and high-risk groups. C-D. Boxplot showing the (C) MHC score and (D) CYT score between low- and high-risk groups. **P* < 0.05, ***P* < 0.001, and ****P* < 0.0001 as calculated using Mann–Whitney U test (B-D).

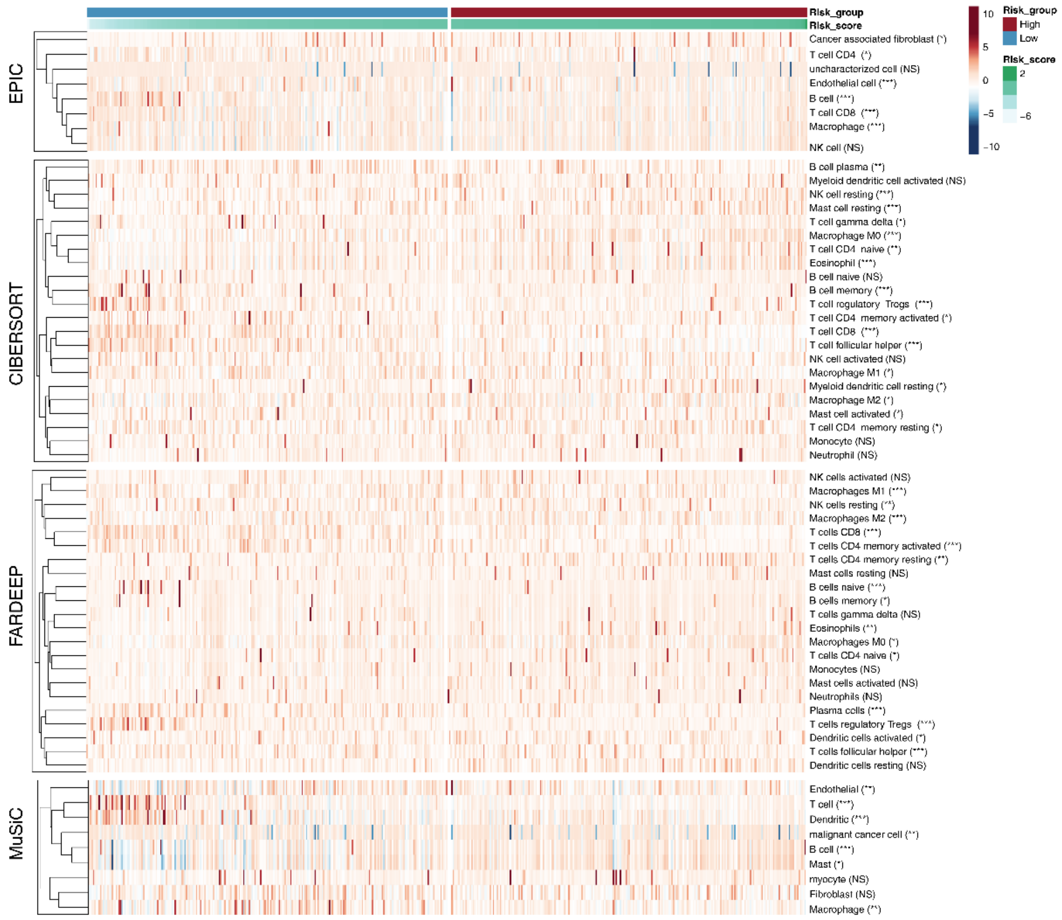


**Figure S5. Heatmap showing the immune cell infiltration levels in low- and high-risk patients evaluated by EPIC, CIBERSORT, FARDEEP, and MuSiC.** The Spearman's correlation significance between cell abundance and risk score was also computed for each cell shown on the right (**P* < 0.05, ***P* < 0.001, and ****P* < 0.0001).
